# Parametric mapping of cellular morphology in plant tissue sections by gray level granulometry

**DOI:** 10.1186/s13007-020-00603-7

**Published:** 2020-05-06

**Authors:** David Legland, Fabienne Guillon, Marie-Françoise Devaux

**Affiliations:** UR1268 Biopolymères, Interactions et Assemblages, INRAE, Nantes, France

**Keywords:** Cellular morphology, Granulometry, Image texture analysis, Mathematical morphology, Parametric mapping, Plant tissue, Quantitative histology

## Abstract

**Background:**

The cellular morphology of plant organs is strongly related to other physical properties such as shape, size, growth, mechanical properties or chemical composition. Cell morphology often vary depending on the type of tissue, or on the distance to a specific tissue. A common challenge in quantitative plant histology is to quantify not only the cellular morphology, but also its variations within the image or the organ. Image texture analysis is a fundamental tool in many areas of image analysis, that was proven efficient for plant histology, but at the scale of the whole image.

**Results:**

This work presents a method that generates a parametric mapping of cellular morphology within images of plant tissues. It is based on gray level granulometry from mathematical morphology for extracting image texture features, and on Centroidal Voronoi Diagram for generating a partition of the image. Resulting granulometric curves can be interpreted either through multivariate data analysis or by using summary features corresponding to the local average cell size. The resulting parametric maps describe the variations of cellular morphology within the organ.

**Conclusions:**

We propose a methodology for the quantification of cellular morphology and of its variations within images of tissue sections. The results should help understanding how the cellular morphology is related to genotypic and / or environmental variations, and clarify the relationships between cellular morphology and chemical composition of cell walls.

## Background

The cellular morphology of plant organs is largely investigated in relation to other physical properties such as shape, size, growth, mechanical properties or chemical composition. In particular, crop species like maize (*Zea mays L.*) are of increasing interest for cattle feeding or for production of bioethanol and biomolecules [1–6]. Several mechanical, biochemical and/or enzymatic processes are involved to transform the raw material, mainly composed of the stem and the leaf cell walls, into energy or fuel. The plant anatomy seems to play a key role in the plant biomass processes, and several investigations on stem histology have been performed to investigate the morphology and the spatial organisation of anatomical structures at various scales: organs, tissues, cells or organites within cells [4, 7–11].

**Fig. 1:**
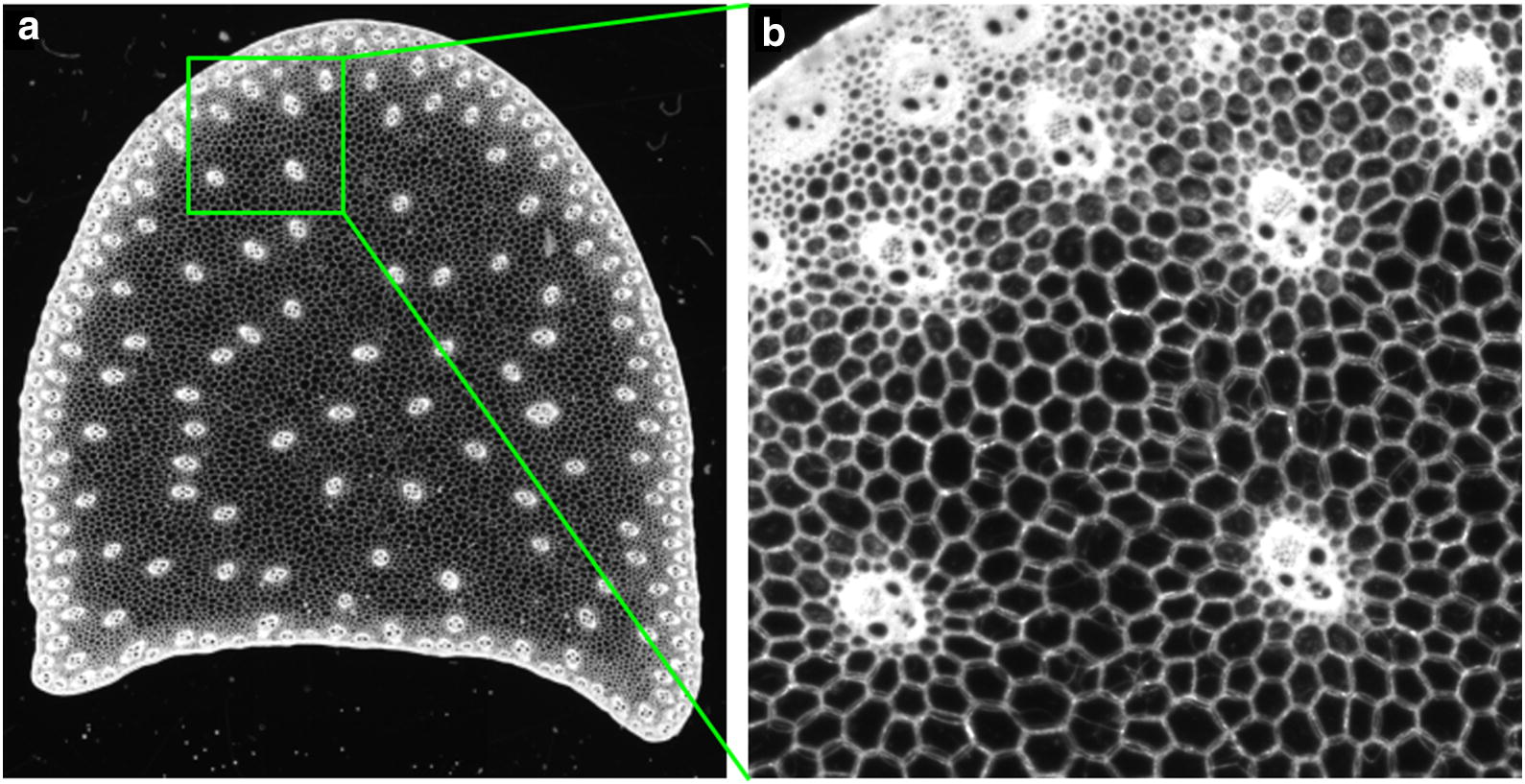
Cellular morphology of a whole tissue slice. **a** Sample image of a cross section of a maize internode showing the variability in cell morphology. **b** Focus on a smaller region close to the epidermis. Parenchyma tissue is composed of large polygonal cells, whereas rind tissue is composed of small cells with thick cell wall

Many properties of plant organs are measured at a macroscopic scale, e.g., mechanical tests on tissues, sensory or chemical analyses, etc. Cell morphology and spatial organisation, on the contrary, are usually investigated using histological approach and imaging tissue sections. Histochemical stainings or spectral imaging furthermore allow to get information on chemical composition [7, 12]. These differences in scale and in size of the field of view make it difficult to establish relationships between the cellular morphology and plant organ properties. In many cases, it is also of interest to consider the variations of a given property within the tissue or within the organ. For instance, the morphology of plant cells may vary depending on the type of tissue, the distance to the epidermis, or the distance to another organ such as a vascular bundle (Fig. 1). A common difficulty is therefore to quantify not only the cellular morphology, but also its variations within the image or the organ.

Image analysis methods are commonly applied to extract meaningful information from images. A classical approach in image analysis is based on the segmentation of one or several regions of interest, and the quantification of features describing each region. Features may be based on the size, the shape, the color... and are usually used as input for a classification process, or for exploring the distribution of the features within a population of objects. The spatial variations of a given feature can be performed by computing a parametric mapping, that consists in a 2D or 3D sampling of estimates of the feature of interest. The feature of interest may be measured directly from the image (gray-level, intensity, or colorimetry mapping), or assessed after computational steps like kernel density estimators for investigating the local density of structures that can be assimilated to points.

Such a strategy is common in various domains. In medical imaging, the intensity of a localized response obtained by Magnetic Resonance Imaging or Positron Emission Tomography is usually interpreted with respect to the related anatomical region. In remote sensing, spectral responses of individual pixels are assessed to quantify land occupation within regions of interest (corresponding to parcels, districts...), or to describe vegetation occupancy across a given territory.

This approach is less common in the context of plant histology. Vilhar et al. [13] estimated cell area within grain sections, using a manual segmentation of cell centroids, and computed parametric maps of cell volume. Pieczywek et al. [14] computed parametric mapping of cell wall fraction obtained from mathematical morphology operators. Variations of cellular morphology within plant organs can be investigated through the distance to the boundary or to a reference structure [15–18]. Concentric regions have also been investigated [7, 11, 14]. However, no generic method for constructing parametric mappings of cellular morphology within plant organs seems to exist.

Image texture analysis is a fundamental tool in many areas of image analysis [19, 20]. Its tasks are mainly classification, segmentation, and image synthesis. A large family of image texture analysis methods are based on gray level co-occurence matrices [21, 22], run length encoding [23], fractal analysis [24–26], or spectral decomposition of the image using Fourier or wavelet bases [27]. Gray-level granulometry is an approach for image texture analysis based on the application of morphological filters (typically opening or closing) of increasing size [28, 29]. Gray-level granulometry results in granulometric curves that can be interpreted in terms of size distribution, making it easier to relate to the physical properties of the studied structures. Another advantage of morphological granulometries is that it is possible to focus on either bright or dark structures in the image, or to consider both of them. This versatility has proven useful for many applications in medical imaging [30–32], material sciences [33], remote sensing [34, 35], or plant sciences [15, 16, 36].

Most applications of image texture analysis concern the indexing or the classification of a collection of images from their global texture, or the segmentation of an image into several regions with homogeneous textures [34, 37]. Few studies investigated the quantitative evaluation of texture features depending on the position [17, 38, 39]. Moreover, most studies use Haralick features, which greatly limits the interpretability of the resulting maps.

The contribution of this work is to present a method that generates a parametric mapping of cellular morphology within images of plant tissues, using gray level granulometry for extracting image texture features. The input image, or a specific region of interest within the input image, is partitioned automatically into an arbitrary number of region of interests. Image texture is assessed by gray level granulometry computed with mathematical morphology filters. The granulometry features computed within each region of interest can be combined with the original partition to generate parametric mapping of the whole image. We performed the partition of the original image by using Centroidal Voronoi Diagram, a particular case of the Voronoi diagram in which the centroid of each region is at the same location as the germ that produces the region [40]. The Centroidal Voronoi Diagram presents a high regularity in both the size and the shape of the regions, making it well adapted for the partitioning of a domain of interest. We present the application of the methods to the description of the spatial heterogeneity of cellular morphology within sections of maize stem internodes observed with macroscopy imaging.

## Methods

**Fig. 2:**
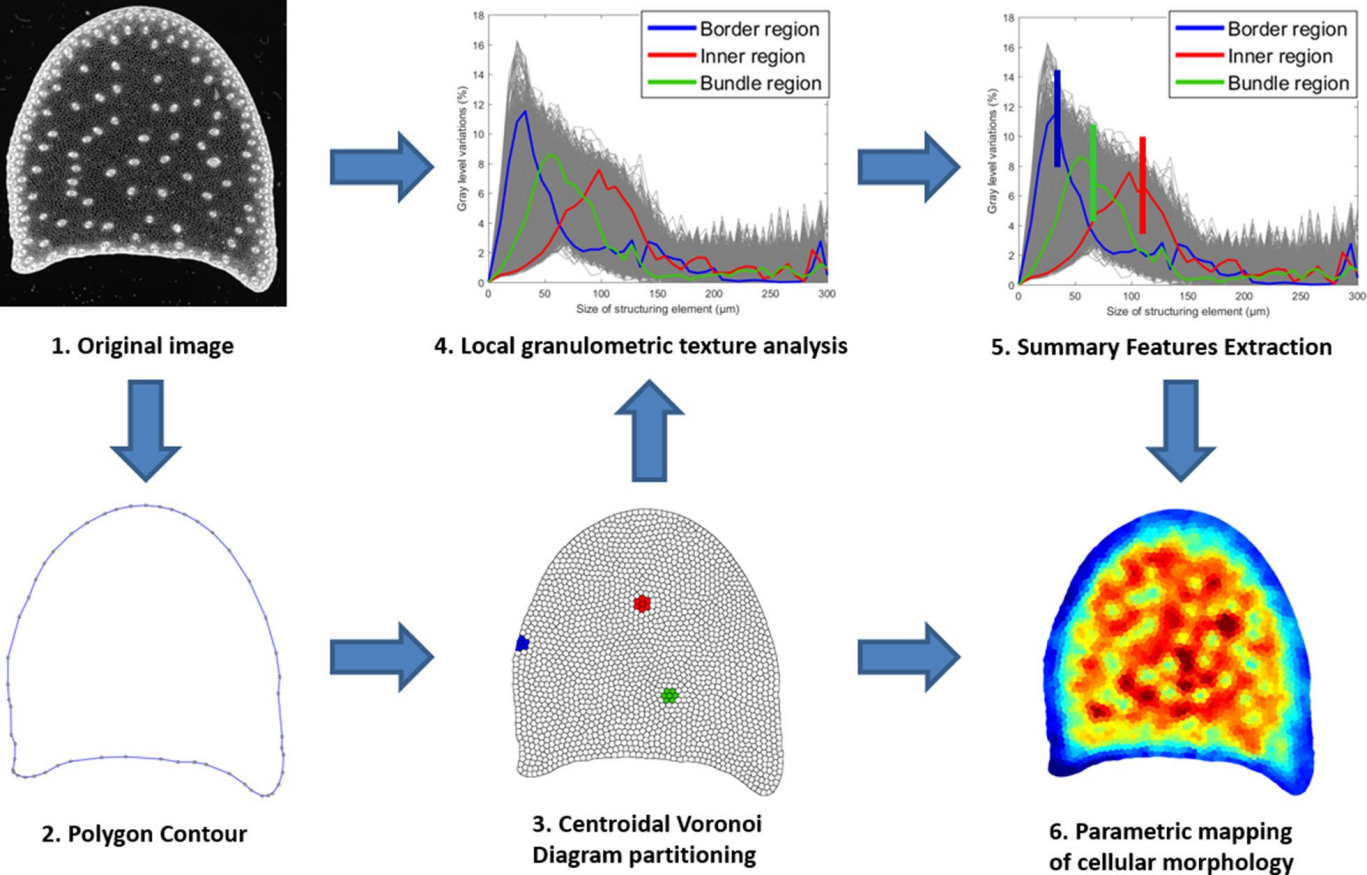
Global workflow for parametric mapping computation. The boundary of the slice is extracted from the original gray-scale image (1), and converted into a polygon (2). The regions of interest are generated within the boundary polygon using the Centroidal Voronoi Diagram (3). Granulometric size distribution for each regions are computed by combining granulometry texture analysis with the regions obtained from the Centroidal Voronoi Diagram (4). For each region, an average size is computed from its granulometric size distribution (5). A parametric mapping is generated using color coding of the average size for each region (6)

This section presents the practical computation of parametric mapping from gray-level granulometry within an arbitrary domain of interest. The global workflow is provided in Fig. 2.

We first recall the definitions of mathematical morphology, then the principles of granulometry analysis, and the practical computation of parametric mapping from texture parameters. The computation of the partioning of the domain of interest using Centroidal Voronoi Diagram is also detailed.

### Mathematical morphology operators

Mathematical morphology provides a number of useful tools for image processing and analysis, based on set theory [29, 41]. A gray level image is considered as a function $f(\varvec{x})$ of a multidimensional point $\varvec{x}$, usually embedded into $\mathbb {Z}^{2}$ or $\mathbb {Z}^{3}$.

The most elementary morphological operators are erosion and dilation. They both are defined depending on a mask with given shape and size, called “structuring element”. Common choices for structuring elements shapes are squares, disks, or line segments. Morphological erosion $\varepsilon _{B}(f)$ and the morphological dilation $\delta _{B}(f)$ of a gray level image *f* consist in computing for each pixel the minimum or the maximum value of the pixels in the neighborhood given by the structuring element. More formally they are defined as:

1 $$\begin{aligned} \left[ \varepsilon _{B}(f)\right] (\varvec{x})&=\min _{\varvec{b}\in B}f(\varvec{x}+\varvec{b}) \end{aligned}$$

2 $$\begin{aligned} \left[ \delta _{B}(f)\right] (\varvec{x})&=\max _{\varvec{b}\in B}f(\varvec{x}+\varvec{b}) \end{aligned}$$

Erosions and dilations are often used in combination. The morphological opening $\gamma _{B}(f)$ of a gray level image *f* is defined as an erosion followed by a dilation:

3 $$\begin{aligned} \gamma _{B}(f) =\delta _{\check{B}}\left[ \varepsilon _{B}(f)\right] \end{aligned}$$

where $\check{B}=\{-b\mid b\in B$} corresponds to the reflection of the structuring element *B* around the origin. Similarly the morphological closing $\phi _{B}(f)$ of the gray level image *f* is defined as a dilation followed by an erosion:

4 $$\begin{aligned} \phi _{B}(f) =\varepsilon _{\check{B}}\left[ \delta _{B}(f)\right] \end{aligned}$$

Morphological closing makes dark objects smaller than the structuring element disappear, whereas morphological opening makes bright objects smaller than the structuring element disappear. Hence, morphological openings and closings are often used as filters to remove bright or dark structures from the image based on a size criterion.

**Fig. 3:**
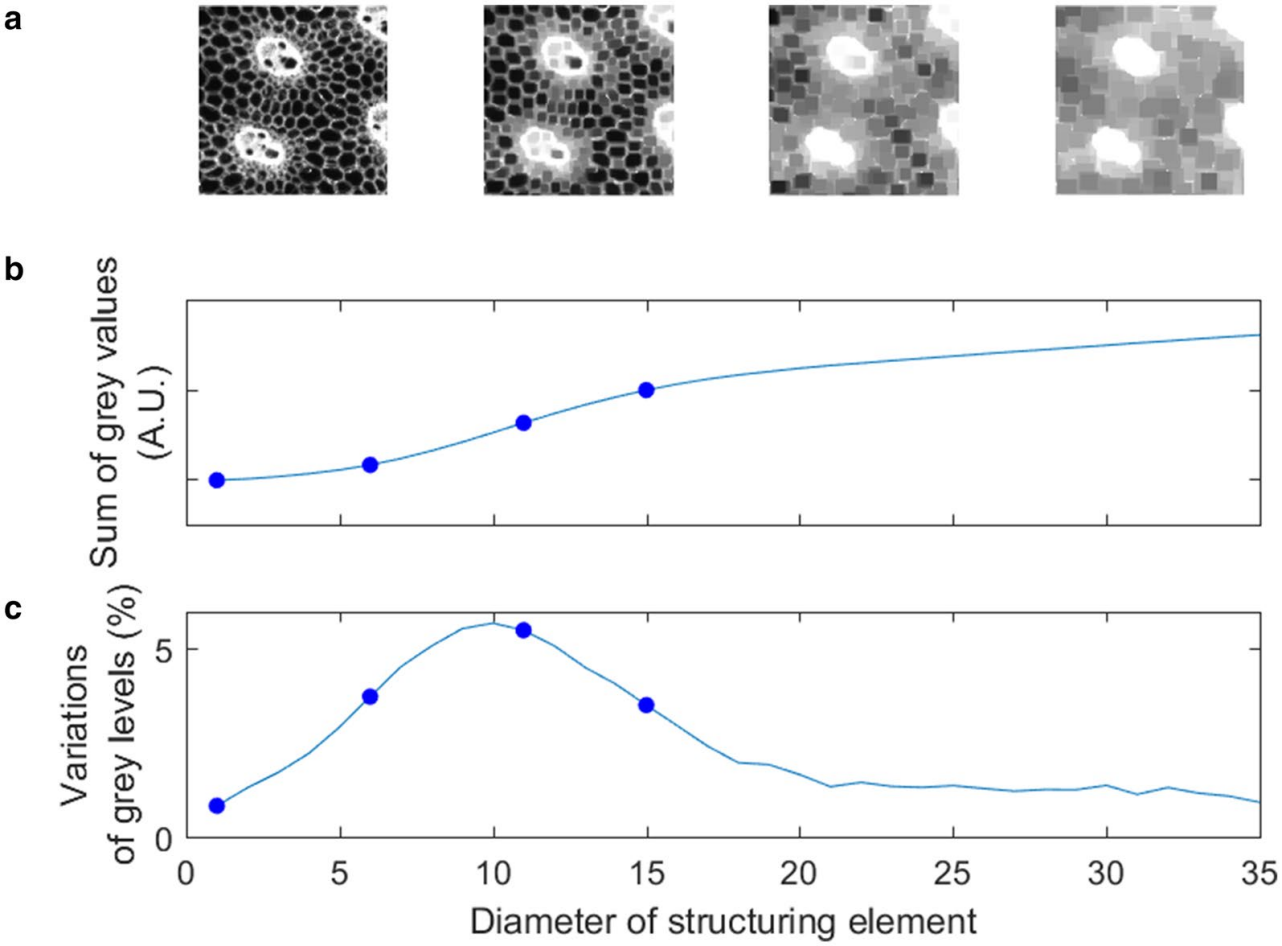
Principle of granulometry. Principle of computation of granulometry curves using morphological closing. **a** Each image corresponds to the result of a morphological closing using increasing sizes of structuring elements. **b** Sum of gray levels in images after application of morphological closings. ** c** Granulometric size distribution, corresponding to the derivative of the curve in (**b**). The dots on (**b**) and (**c**) curves correspond to the stages shown in (**a**)

**Fig. 4:**
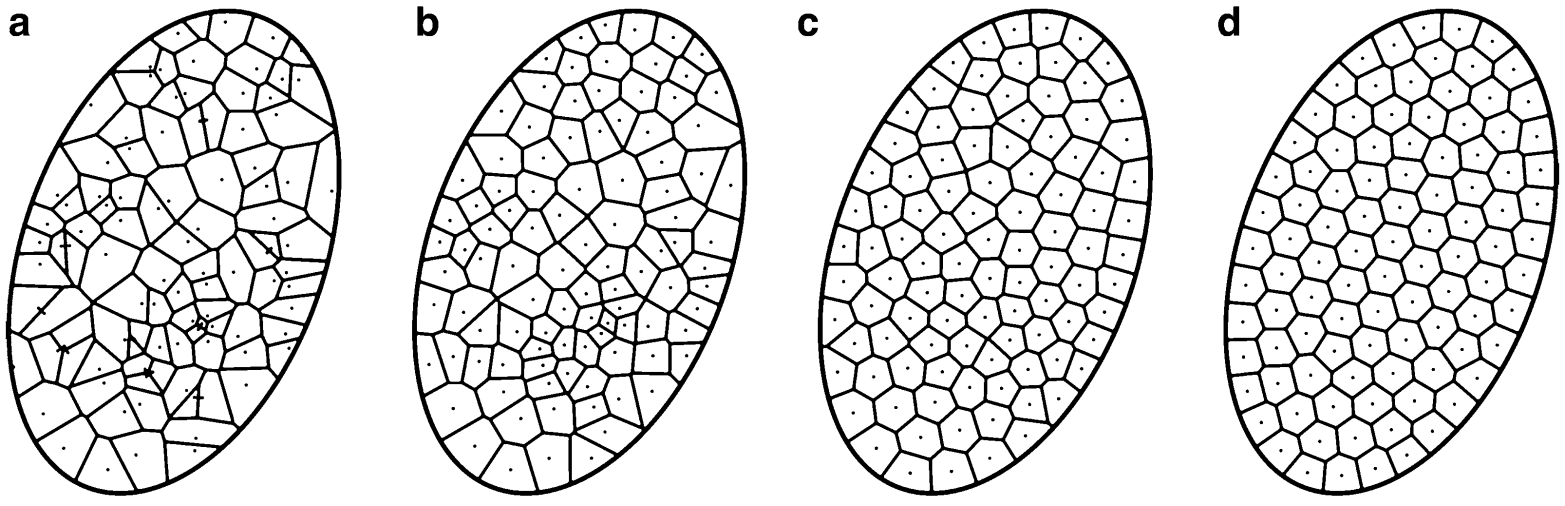
Computation of Centroidal Voronoi Diagram. Several iterations of the computation of centroidal voronoi regions computed within an ellipse domain. Initial Voronoi Diagram (**a**), resulting diagram after (**b**) 1, (**c**) 10 and (**d**) 100 iterations. At 100 iterations, stability was reached

**Fig. 5:**
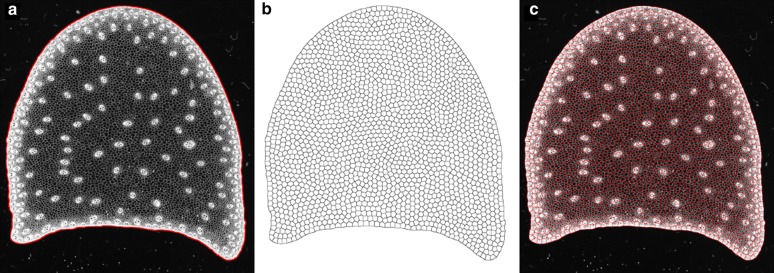
Partitioning of the stem section. The maize stem cross-section is partitioned into a large number of regions by using the Centroidal Voronoi Diagram. **a** Original image with boundary super-imposed. ** b** Result of the partition. ** c** Result of the partition super-imposed on the original image

**Fig. 6:**
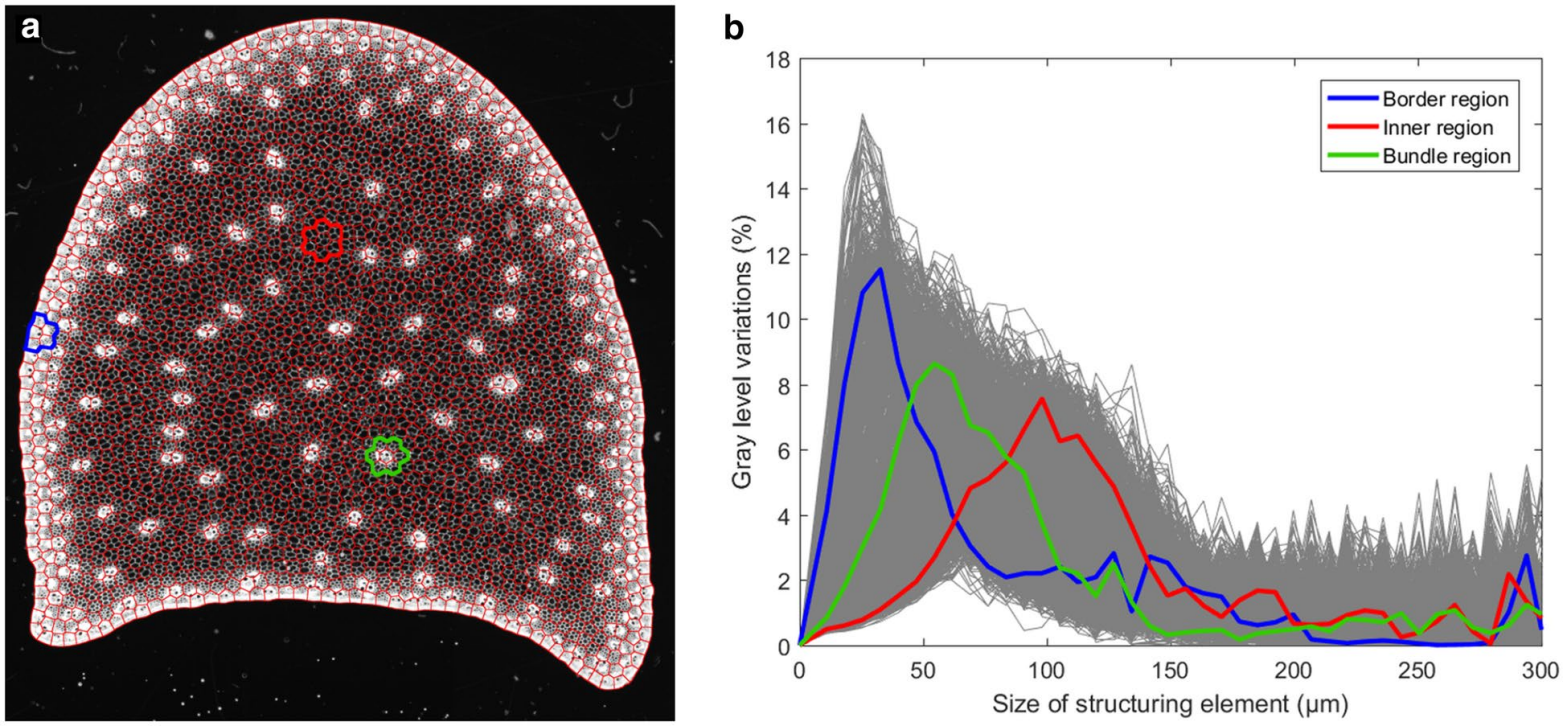
Computation of granulometric curves for different regions of interest. ** a** Result of the partition super-imposed on the original image. Three thickened polygons hightlight regions corresponding to specific tissues: parenchyma (red), rind (blue), vascular bundle (green). ** b** Computation of granulometric curves for each region of interest. The complete collection of granulometric curves is shown as gray. The granulometric curves corresponding to the highlighted regions are shown in thick colored lines

**Fig. 7:**
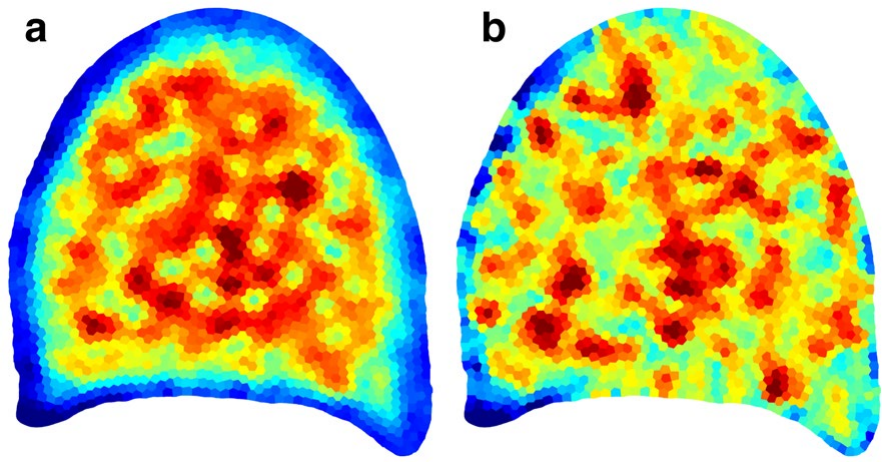
Parametric mapping of summary features. Parametric mapping of summary features obtained from granulometry curves within each region of interest. ** a** Parametric mapping of the geometric mean size. Large values are located within the parenchyma, whereas border regions presents small value. ** b** Parametric mapping of the standard deviation of granulometry curve within each region. Blue colors correspond to low values, corresponding to homogeneous cell sizes, red colors corrrespond to high values, corresponding to heterogeneous cell sizes within the region

### Granulometry and granulometric size distribution

The notion of granulometry and granulometric functions were first introduced by Matheron [42] as tools to extract size distributions from binary images, and were later extended to gray level images. The principle of gray level granulometry consists in applying morphological operators, ususally openings or closings, with various sizes of structuring element, and in measuring the quantity of changes between two successive steps to obtain a distribution of the size of the structures in the image [29, 32, 37].

**Fig. 8:**
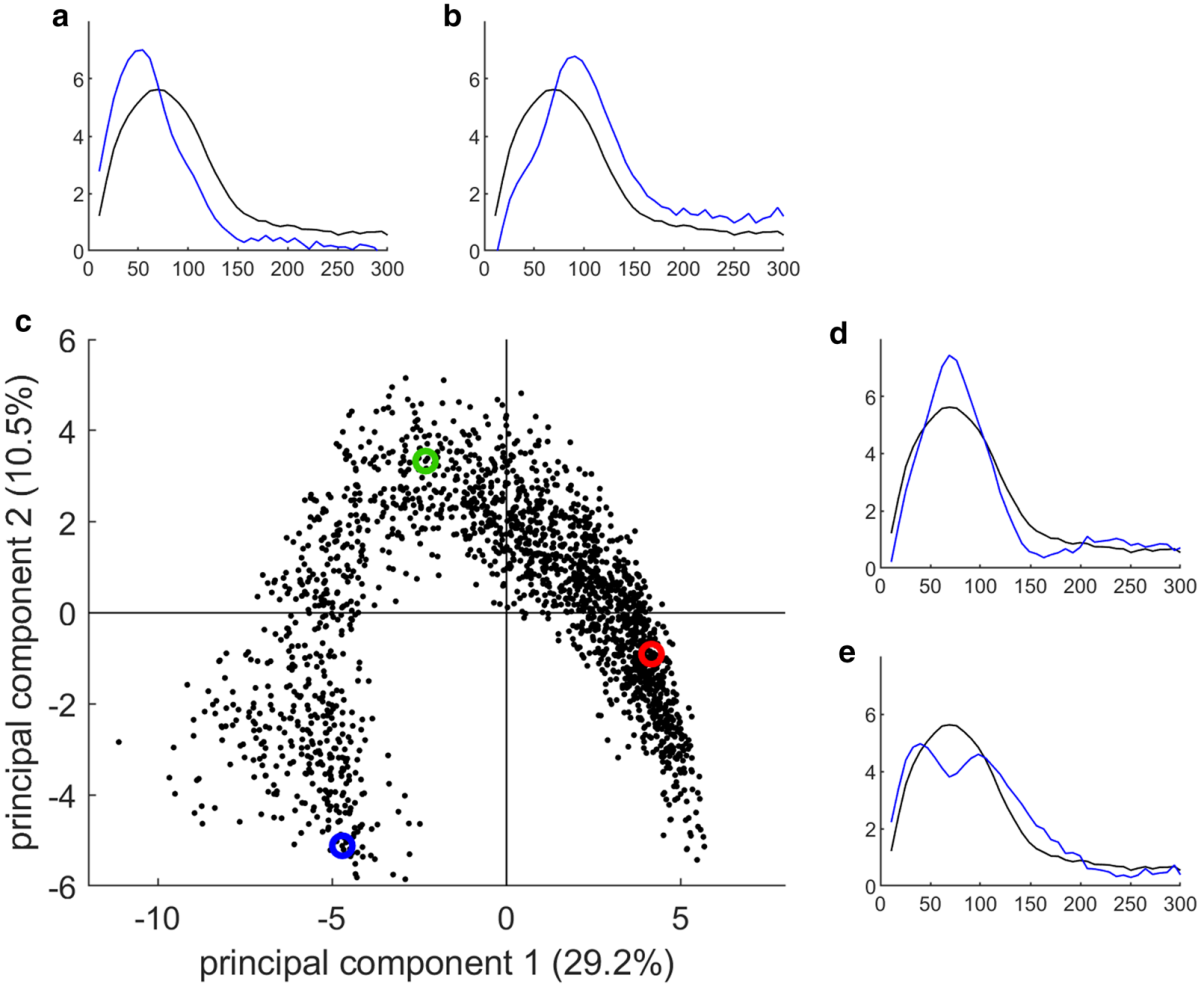
Statistical analysis of the granulometry curves. Statistical analysis of the whole set of granulometry curves. Panel** c** plots each region of interest along the first two axes of the principal component analysis. Colored dots correspond to sample regions shown in Fig. 6. (**a**–**e**) Synthetic curves curves corresponding to the reconstruction of the average curve $\pm 2\sqrt{\lambda _{i}}$ the loadings are shown along corresponding axes (blue curves), exhibiting differences with the average size distribution (black curve). On the first axis, curves **a**, ** b** hightlight differences in the position of the mode of the distribution. On the second axis, curves (**d**, ** e**) hightlight the differences of proportion of cells with average size

**Fig. 9:**
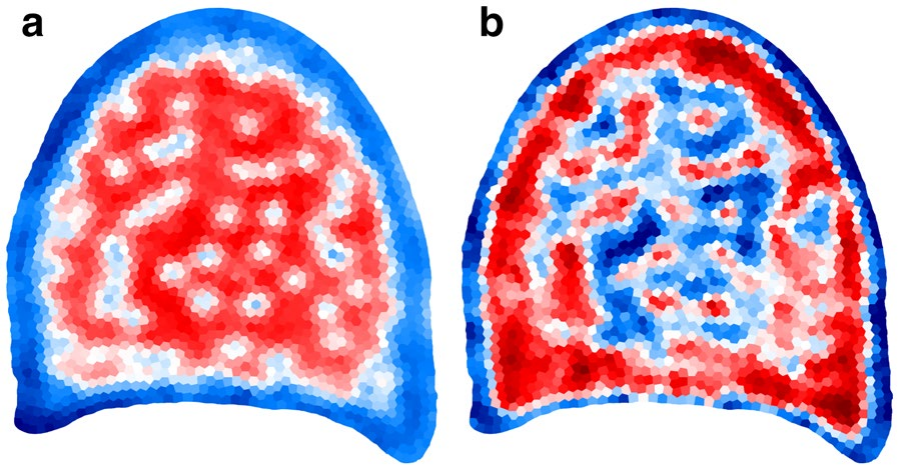
Parametric mapping of PCA scores. Color representations of parametric mappings obtained from granulometry curves within each region of interest. **a** Parametric mapping of the scores along the first principal component. **b** Parametric mapping of the scores along the second principal component. Red colors correspond to positive values, blue colors to negative values, and bright colors to values close to zero

The computation of granulometric curves requires a series $B_{\lambda _{i}},{}_{i=1,\ldots n}$ of structuring elements of increasing sizes, such that $i<j\implies B_{\lambda _{i}}\subseteq B_{\lambda _{j}}$. Morphological openings [resp. closings] are performed using each structuring element. Increasing the size of the structuring element makes bright [resp. dark] structures progressively disappear. Figure 3 presents an example of several gray-level morphological closings applied successively on a portion of image of plant tissue.

Each step is summarised by the image volume curve $V_{i}$, that corresponds to the sum of the pixel gray level values in the corresponding processing step. When using morphological closing, the image volume curve is given by:

5 $$\begin{aligned} V_{i} =\sum \phi _{B_{\lambda _{i}}}(f) \end{aligned}$$

The curve $V_{i}$ is monotonically increasing, starting from $V_{0}$, the initial sum of gray level values in image, and tending to $V_{\infty },$ the sum of gray values after an closing of arbitrarily large size (Fig. 3). The granulometric size distribution $G_{i}$, or pattern spectrum, corresponds to the derivative of the image volume curve normalized by the initial and final values $V_{0}$ and $V_{\infty }$:

6 $$\begin{aligned} G_{i} =\frac{V_{i+1}-V_{i}}{V_{\infty }-V_{0}} \end{aligned}$$

The granulometric size distribution measures the differences between successive closings (Fig. 3). The original curve $V_{i}$ of the gray level volumes may be seen as the inverse of the cumulative size distribution. Granulometric size distribution computed from gray-level images can be compared to usual granulometric distributions except that they are calculated taking gray level variations into consideration. The position of the peak in granulometric size distribution (Fig. 3, bottom) corresponds to the typical size of the dark structures in the image (here, cells). The width of the peak can quantify the heterogeneity of structure sizes in the image: the broader the peak, the more heterogeneous the size of structures.

Granulometric size distributions can be interpreted as usual size distributions, and can be summarized by an adequate summary statistics. The central moments of the distribution are commonly used [37, 43], but other choices can be made: the statistical mode, the median value... The geometrical mean appears as a good compromise that takes into account the whole distribution, and whose value is closer to the center of the peak than the mean or the median. The geometrical mean of a granulometric size distribution is expressed as follow:

7 $$\begin{aligned} m_{G}= \exp \left[ \sum _{i}\log \left( \lambda _{i}\right) \cdot G_{i}\right] \end{aligned}$$

where $\lambda _{i}$ corresponds to the size of the structuring element at step *i* and $G_{i}$ to the fraction of image strucure that disappear at step *i*. The value $m_{G}$ is called here “gray level mean size” to indicate that it is based on gray level measurements.

### Local granulometric size distributions

In practical applications of image texture analysis it can be useful to restrict the analysis to a region of interest corresponding to an enclosing structure of interest, such as a biological organ (in biomedical imaging), or a specific geographic territory (in remote sensing). The concept of local granulometric size distribution was developped by Dougherty and his coworkers [37, 43, 44]. As with the original global granulometry, morphological openings (or closings) with structuring elements of increasing sizes are applied to the whole image. However, the size distributions are computed locally in a given region of interest *R*. The local granulometric size distribution $G_{i,R}$ within region *R* is defined as follow:

8 $$\begin{aligned} G_{i,R} =\frac{V_{i+1}(R)-V_{i}(R)}{V_{\infty }(R)-V_{0}(R)} \end{aligned}$$

where $V_{i}(R)$ is the sum of the gray levels of $\gamma _{B_{\lambda _{i}}}(f)$ or $\phi _{B_{\lambda _{i}}}(f)$ computed within the region of interest *R*.

The granulometric size distributions computed in a collection of regions of interest are all computed with the same set of sizes $\lambda _{i}$, and may also be analysed and exploited with the help of multivariate analysis. An application using the principal components analysis is presented in the Results section. The computation of gray level mean size $m_{G}(R)$ in a given region of interest *R* is defined similarly to the global case, by taking into account the local granulometric size distribution $G_{i,R}$.

A possible choice for the regions of interest *R* is a square window centered around each pixel, thus generating a granulometric size distribution for each pixel [30, 44]. Such a strategy may however result in a large variability in granulometric size distributions, as each curve is computed in a region comprising only one pixel. Moreover, computing granulometric size distributions for each pixel may lead to high memory requirements when applied to large images and using gray level granulometries with a large number of structuring element sizes. Finally, the computation of granulometric size distributions within arbitrary domains may be complicated by the management of edge effects. Consequently, we generated partitions of the domain by using Centroidal Voronoi Diagram, resulting in regions with a compact shape and similar sizes

### Generic partitioning using Centroidal Voronoi Diagram

The Voronoi Diagram of a point set (called the germs) is a fundamental geometric structure that partitions the space into elementary regions of influence [45]. Given an open domain $\Omega$ of $\mathbb {R}^{d}$, and *n* different germs $\varvec{g}_{i};i=0,1,...,n-1$, the Voronoi Diagram can be defined as a set of *n* distinct regions $R_{i}$ such that:

9 $$\begin{aligned} R_{i}= \left\{ \varvec{x}\in \Omega |d(\varvec{x},\varvec{g}_{i})<d(\varvec{x},\varvec{g}_{j}),\,j=0,1...,n-1,j\ne i\right\} \end{aligned}$$

where *d* is a distance function, usually the Euclidean one. An example of a Voronoi Diagram constrained to an elliptical domain is shown in Fig. 4a. The centroidal Voronoi diagram is a special case of the Voronoi diagram, that exhibits the property that the centroid of each region corresponds to the germ that produces the region [40]. Given a uniform density function, the centroid of the region $R_{i}$ is obtained by:

10 $$\begin{aligned} \varvec{c}(R_{i})= \frac{\int _{R_{i}}\varvec{x}d\varvec{x}}{\int _{R_{i}}d\varvec{x}} \end{aligned}$$

A centroidal Voronoi Diagram with *n* regions can be computed by the Lloyd algorithm [40]. It consists in the following steps :

1. Generate *n* random points corresponding to the initial germs within the domain of definition,
2. Generate the Voronoi Diagram coresponding to the germs,
3. Compute the centroid of each region of the Voronoi Diagram,
4. Set the centroids as new germs,
5. Iterate from (2) until stability.

Figure 4 shows several steps of the algorithm. Germs were generated following a Poisson distribution within reference region (Fig. 4a). A large heterogeneity in the size of the polygonal regions may be noticed, as well as the presence of many elongated regions. Starting from the initial germs, the Lloyd algorithm was applied, by successively computing the centroid of each polygonal domain, and computing the Voronoi diagram of the resulting points. As the number of iterations increases, the regions become more and more regular: they are more homogeneous in size, and are less elongated.

### Practical implementation

The granulometric curves were computed by applying sequences of morphological closing operation using structuring elements of increasing sizes. In order to reduce the overall computation time, morphological operations were applied on the whole image, and the sums of gray levels were computed on each region within each step. The whole collection of granulometric curves was computed after all morphological operations were performed. The whole image processing workflow was implemented within the Matlab software (The Mathworks, Natick, MA), using the Image Processing Toolbox. The functions developed for parametric mapping of granulometry curves have been integrated into the MatImage toolbox.^1^

The computation of Centroidal Voronoi Diagram was performed by using Monte-Carlo approach for computing the centroids. The principle is to generate a collection of query points, identify the closest germ to each query point, and compute the centroid of the query points associated with a given germ. The generation of query points within an arbitrary polygonal domain can be performed using rejection sampling, making it easy to implement even for domains with complex geometries. Functions for computing Centroidal Voronoi Diagrams were implemented with help of the MatGeom library.^2^

## Results

The whole methodology was applied for the computation of parametric mapping of cell morphology within images of maize internodes cross-sections. Maize is a model species for the study of cell wall chemical composition and degradability, with a major focus on the production of bio-fuels. In addition to the variability of the composition of cell types, the morphology of cells, as well as its heterogeneity within the tissues, should be taken into account to better understand plant degradation or mechanical properties.

### Imaging of plant tissues

The same plant materials as in [7] were used. Images were obtained using the same general workflow as in [8], except that the whole stem section could be observed. Maize stem sections were obtained using a vibrating razor blade, using a section thickness equal to 200 microns. This thickness was found experimentally to be large enough to maintain the physical integrity of the slice, and small enough to avoid the superposition of too many cell layers. Slices were imaged using a specifically designed device consisting in a controlled-light digital camera and a specific control software [15]. A sample result of image acquisition is shown on Fig. 1. Different structures can be distinguished:

- The external region of the stem with small cells and vascular bundles that appeared as a white structure on the whole image. It is called the rind
- The vascular bundles that are distributed in all the sections
- The parenchyma cells that are found between the vascular bundles.

The resolution of about 3.6 micrometers makes it possible to distinguish small cells as well as to evidence the variability in cell morphology through the section.

### Determination of regions of interest

The binary mask corresponding to the stem section was first segmented using gray level threshold and image filtering based on a combination of morphological openings and closings. The contour of each section was extracted and converted to a polygon by chaining adjacent contour pixels [8] (Fig. 5). To reduce computation time of posterior computation steps, the number of vertices of each polygon was reduced by mean of the Douglas-Peucker algorithm [46]. A tolerance distance of two pixels was used.

A collection of regions of interest $R_{i}$ was generated by computing the Centroidal Voronoi Diagram within the contour of the section. The number of regions was set to 2000, as it was found to be a good compromise between the spatial resolution and the size of the regions. The number of iterations was set to 100. After convergence was reached, an image of region labels was generated by computing the index of the region of influence of each germ: for each pixel of the resulting map, the closest germ was identified, and its index was used as label. A complete partition of the domain corresponding to the stem section could be obtained (Fig. 5). The resulting regions exhibited great regularity, with similar areas and low elongation values (Fig. 5).

### Computation of granulometric curves

The collection of granulometric curves within each region of interest was obtained as described by Eq. 8. When the size of the regions is small, the resulting granulometric curves tend to be noisy, exhibiting many small peaks. Consequently, the granulometric curve of each region $R_{j}$ was computed by considering the variations of gray levels in a larger region $R'_{j}$ corresponding to the union of $R_{j}$ with all its surrounding regions. The Region Adjacency Graph (RAG) of the image of region labels was used to quickly identify the neighboring regions. The RAG of a labeled image is a graph whose vertices correspond to the regions and whose edges link neighbor regions.^3^ Sample regions of interest corresponding to contrasted tissue types are shown on Fig. 6. The equation for computing granulometric size distribution is therefore given by the following:

11 $$\begin{aligned} G_{i,R_{j}} =\frac{V_{i+1}(R'_{j})-V_{i}(R'_{j})}{V_{\infty }-V_{0}} \end{aligned}$$

The whole collection of granulometric curves was transformed into a data table with *n* rows corresponding to the regions of interests, and *p* columns corresponding to the different sizes of structuring elements. The collection of granulometric curves is shown in Fig. 6. A large variability can be noticed. The curves corresponding to three specific regions are highlighted. The curve corresponding to the first region (blue), located in the rind on the border of the section, shows a thin peak for small (around 20–30 microns) sizes of structuring element. This can be interpreted by the presence of a majority of small cells in the periphery of the section. On the contrary, the curve corresponding to the second region (red), located in the middle of the section with only parenchyma cells, shows a large peak around 100 microns. This corresponds to the presence of large cells within the inner tissues of the section. The third region (green) corresponds to a vascular bundle. The associated curve shows a peak around 50 microns that can be interpreted as an increased proportion of cells with intermediate diameter. The typical sizes obtained from granulometric curves were in accordance with visual inspection of images, and with results from the litterature [4, 11]

### Mapping of summary features

In order to facilitate the exploration of variations of granulometric along the slices, a natural approach is to compute a summary statistic for each curve, and display this statistic using an adequate color-coded representation. Figure 7 shows two examples of parametric mappings corresponding to the geometric mean, and the standard deviation, both of them computed from each granulometric size distribution.

The parametric mapping of the geometric mean is shown on Fig. 7a. Blue colors correspond to regions with small geometric mean, i.e. to regions containing mostly small cells. Such regions are mainly located on the periphery of the section. The regions corresponding to the parenchyma are associated with large geometric mean size (represented as red color). Regions located around vascular bundles present smaller geometric mean size compared to the regions located in the parenchyma.

The parametric mapping of the standard deviation is shown on Fig. 7b. Low values (blue and green colors) can be observed at the periphery of the slice, corresponding to homogeneous populations of small cells, and in parenchyma regions, corresponding to homogeneous populations of cells with large diameters. The regions with high values of standard deviations are mostly found around vascular bundles, corresponding to an increase of the diversity of the cell sizes.

### Principal component analysis of granulometric size distributions

The collection of granulometric curves may also be analysed globally by principal component analysis [47]. Principal component analysis is a multidimensional data processing that reveals the similarities between samples by taking all variables into account. Similarity maps, drawn from the principal component scores, are used to compare the samples and to identify clusters of similar samples. Applied to ordered signals such as granulometric size distributions, synthetic size distributions can be reconstructed from principal component loadings, highlighting changes from the average size distribution.

A result of principal component analysis is presented on Fig. 8. A large variability can be observed, together with a strong structuration of the data. The first two principal components capture around 40% of the variability. This relatively small value indicates that the total variability within the curves is much larger than the projection on two dimensions. The shape of the point cloud formed by the score plot can be interpreted as a strong structuration of the data. The three colored dots corresponds to the sampled regions shown Fig. 6, corresponding to the border of the section (in blue), to the inner region, mostly composed of parenchyma cells (in red), and to a vascular bundle (in green). The three dots are well separated on the similarity map formed by the first two principal components.

Synthetic size distributions for the first two principal components are represented along each axis as blue curves, exhibiting differences with the average size distribution represented with a black curve. The average size distribution is rather smooth, presenting a bulb around 70 microns. The mode of the bulb can be interpreted as the typical diameter of the cells within the slice.

The synthetic curves on the first principal component (representing 29.2% of total inertia) correspond to an increase in the quantity of cells with diameter 100 microns (positive direction), or an increase in the quantity of cells with diameter around 50 microns (negative direction). It hence corresponds to variations in the average cell size in the corresponding region. A parametric mapping can be obtained from the scores of the first principal component, and using adequate color representation (Fig. 9a). As the first component can be related to the average size of cells, the regions colorized in red correspond to large cell, whereas the regions colorized in blue correspond to small cells.

There is an obvious similarity between the geometric mean values (Fig. 7a) and the scores of the first principal component (Fig. 9a). This appears rather logical, as the first principal component was interpreted as variation in the cell size within the region. The values obtained from geometrical mean, however, can be interpreted more easily in terms of cell size. Such measurement is of interest if an actual value of cell size is required to build mechanical model of morphogenesis, for example.

The analysis of the second principal components (representing 10.5% of total inertia) may be performed similarly be reconstructing synthetic granulometric curves representing variations around the mean curve. A similar representation can be obtained from the scores along the second principal component (Fig. 9b). The synthetic size distribution indicates that the second principal component discriminate regions with a proportion of cells with average diameter (around 70 micrometers), and regions with low proportion of average cells but with either smaller or larger cells. The inspection of the corresponding parametric mapping shows that regions with low value on second component correspond either to parenchyma regions (composed of a majority of large cells), or at the periphery in the rind (composed of a majority of small cells). A region below the rind that corresponds to homogeneous cells with 70 micrometer diameter is revealed on this map. The examination of further components indicates differences involving several populations of cell sizes, making the interpretation more difficult (data not shown).

## Discussion

The proposed method allows to compute parametric mapping of texture features from plant tissue sections. The method is based on gray level mathematical morphology for computing granulometric curves, and on the Centroidal Voronoi Diagram for computing a partition of the image. The granulometric curves can be interpreted in terms on size distributions of cells within tissue.

The use of the Centroidal Voronoi Diagram allows for computing the parametric mapping either for the whole image, or within a specific domain of arbitrary shape, which is of interest in practical applications. It is also possible to adjust the quality of the parametric mappings, by changing the number of regions used to build the Centroidal Voronoi Diagram. While dependent on the application, the optimum number is a compromise between the spatial resolution of the mapping on one hand and the representativity of the information within the regions on the other hand. Increasing the number of regions results in mappings with better spatial resolution, but the large number of granulometric curves may be more complex to analyse, or require more time to compute. The size of the regions use for measurements may also be too small to capture a representative number of cells. The resulting granulometric curves would be more noisy, and the parametric mappings would show large differences between neighbor regions. On the contrary, using a smaller number of regions generates granulometric curves that are more representative of the cell morphology within the region, at the price of loss of spatial resolution of the resulting mapping. Using fewer regions may also be as strategy to quickly check the variations in cell morphology at the scale of the organ or of the sample. In this study, the number of regions that was chosen resulted in regions of interest with diameters approximately equal to 500 microns. As the typical size of the largest cells was around 100 microns, the regions were large enough to contain at least several cells. The resulting granulometric curves were regular enough to provide accurate estimates of average cell sizes or standard deviations.

When increasing the number of regions, the number of granulometric size distributions to be analysed can be large. The use of summary features such as the mean (arithmetic or geometric) or the standard deviation provides a convenient way to assess the main information within the data set. Exploratory data analysis is a powerful alternative that allows to explore the whole collection of granulometric curves, and to represent graphical spatial heterogeneity of principal components. In particular clustering methods can be envisioned to identify distinct tissue types.

The whole workflow should easily be applicable to other images of plant tissues, as long as cells or other structures of interest can be distinguished. The limitations may come from the resolution of the imaging system. In this study, the resolution of 3.6 microns was compatible with the observation of the cell morphology (10–20 microns for small cells). The resolution was however not sufficient to quantify the thickness of cell walls by computing opening-based granulometries, for example. Apart from plant tissues, the whole methodology is rather generic, and can be applied to a variety of domains. In particular, the parametric mapping of textural changes from medical histology sections should be possible.

Several extensions of the proposed method may be envisioned. A first extension could be to locally quantify the anisotropy, by means of directional granulometries [48, 49]. The computation of parametric mappings obtained from attribute granulometries [50–53], for example area opening, should also provide meaningful results, as the resulting granulometries can be made independent from the shape of the structuring element The use of non-flat structuring elements may be envisioned, at the cost of an increased computational complexity.

With the generalisation of 3D image acquisition systems such as tomography, the adaptation of the method to 3D parametric mappings would also be of interest [10]. The computation of Centroidal Voronoi Diagrams is mainly based on distance computation, well defined for 3D as well, and efficient implementations of mathematical morphology are now easily available for 3D images [54, 55].

## Conclusions

We have presented a method for computing parametric mapping of texture features obtained from gray-level granulometries. It is based on gray level mathematical morphology for computing granulometric curves, and on the Centroidal Voronoi Diagram for computing a partition of the image. The methodology was applied to quantify the variations of cellular morphology within plant tissue sections. The resulting parametric maps of cellular morphology were found to be in adequation with visual inspection of images, and with quantative results obtained from previous studies.

The quantitative results provided by the method are expected to facilitate the comparison of plant obtained from different genotypes and/or with growing with different environmental conditions. By providing spatial localisation of the measurement, the changes of morphology for specific tissue or tissue regions type will be facilitated.

Another advantage of the method is the possibility to jointly analyse cellular morphology features with local chemical composition. The parametric maps of cellular morphology can be superimposed to local measurements of cellular compositions obtained by histochemical staining [1, 7] or spectral imaging [56].

Further work concern the parametric mapping of local anisotropy of cellular morphology, and the joint analysis of parametric maps of cellular morphology with features obtaines from other acquisition devices (histochemical imaging, fluorescence imaging...). The integration and the fusion of imaging modalities from multiple sources and at multiple scales [8, 57, 58] should help reveal relationships between morphometry and chemistry of plant tissues.

## Data Availability

The datasets used and/or analysed during the current study are available from the corresponding author on reasonable request.

## Footnotes

1 https://github.com/mattools/matImage.

2 https://github.com/mattools/matGeom.

3 https://fr.mathworks.com/matlabcentral/fileexchange/16938-region-adjacency-graph-rag.
